# QuEChERS净化结合超高效液相色谱-串联质谱法测定精油类化妆品中13种禁用生物碱

**DOI:** 10.3724/SP.J.1123.2025.10009

**Published:** 2026-06-08

**Authors:** Zhen LIU, Yumei WANG, Jianbin PAN, Lingli ZONG, Yuhan SONG, Xiaojie SUN, Hongyuan CHEN

**Affiliations:** 1.南京市食品药品监督检验院，江苏 南京 211198; 1. Nanjing Institute for Food and Drug Control，Nanjing 211198，China; 2.南京大学化学化工学院，江苏 南京 210023; 2. School of Chemistry and Chemical Engineering，Nanjing University，Nanjing 210023，China

**Keywords:** QuEChERS净化, 超高效液相色谱-串联质谱法, 精油类化妆品, 禁用生物碱, QuEChERS purification, ultra performance liquid chromatography-tandem mass spectrometry （UPLC-MS/MS）, essential oil-based cosmetics, prohibited alkaloids

## Abstract

生物碱是一类广泛存在于植物中的天然化合物，部分生物碱因其潜在的毒性已被明令禁止在化妆品中添加。随着精油类化妆品的普及，其植物来源成分可能引入禁用生物碱，带来安全隐患，因此建立快速、准确的分析方法对产品安全风险评估具有重要意义。本文开发了一种基于QuEChERS前处理结合超高效液相色谱-串联质谱（UPLC-MS/MS）的分析方法，用于同步测定精油类化妆品中13种禁用生物碱。样品经含0.1%甲酸的甲醇溶液超声提取，QuEChERS粉末净化后无需浓缩，直接进样分析；采用Waters ACQUITY UPLC HSS T3色谱柱（100 mm×2.1 mm， 1.8 μm）分离，以乙腈和0.1%甲酸水溶液为流动相进行梯度洗脱，电喷雾电离正离子模式（ESI^+^）和多反应监测（MRM）进行定性与定量分析。该方法在0.2~50 ng/mL范围内线性关系良好，相关系数均大于0.99，检出限（LOD）为1~4 μg/kg，定量限（LOQ）为2~10 μg/kg。在3个加标水平下的平均回收率为83.9%~119.1%，相对标准偏差（RSD）不超过7.3%（*n=*6）。为考察方法的实用性和适用性，将该方法用于50批市售精油化妆品中13种禁用生物碱的检测，样品包括15批宣称婴幼儿使用或儿童产品以及35批成人用精油产品。结果显示，所有样品中均未检出本方法所涵盖的13种禁用生物碱。本方法操作简便，灵敏度高，重复性好，适用于精油类化妆品中多种禁用生物碱的高通量筛查与检测，其中欧夹竹桃苷检测方法的建立为该领域检测技术的发展提供了新的思路，为化妆品质量控制和消费者安全提供了可靠的技术支撑。

## Abstract (en)

Alkaloids represent a class of naturally-occurring nitrogen-containing compounds widely distributed across diverse plant species. Owing to their well-documented potential to induce adverse effects on human health， certain alkaloids are explicitly prohibited from being used in cosmetic formulations. The escalating global popularity of essential oil-based cosmetics， which commonly incorporate complex botanical extracts， presents a potential avenue for the inadvertent introduction of these prohibited substances. Consequently， the development of robust， sensitive， and efficient analytical methods for their monitoring is of utmost significance for consumer safety and regulatory compliance. This study devises a reliable， high-throughput approach for the simultaneous determination of 13 prohibited alkaloids in essential oil-based cosmetics. It integrates optimized QuEChERS sample preparation with ultra performance liquid chromatography-tandem mass spectrometry （UPLC-MS/MS）. The sample preparation procedure was meticulously designed to maximize efficiency and minimize analyte loss. Chromatographic separation was accomplished on a Waters ACQUITY UPLC HSS T3 column （100 mm × 2.1 mm， 1.8 μm） maintained at a constant temperature of 30 ℃. The mobile phase was composed of （A） acetonitrile and （B） 0.1% （volume fraction） formic acid aqueous solution. A gradient elution program was implemented at a stable flow rate of 0.3 mL/min according to the following profile： an initial 5， a linear increase to 15 （0-2 min）， a rapid rise to 70 （2-4.5 min）， followed by an immediate reversion to the initial 5 （4.5-5.5 min）， which was maintained for re-equilibration until 7.0 min. The injection volume was 5 µL. Detection and quantification were conducted using a triple quadrupole mass spectrometer equipped with an electrospray ionization （ESI） source operating in positive ion mode （ESI^+^）. Data acquisition was carried out in the multiple reaction monitoring （MRM） mode to ensure superior specificity and sensitivity. For each of the 13 alkaloids， two specific ion transitions were monitored： one for quantitative analysis and the other for confirmatory identification. The method was rigorously validated in accordance with accepted analytical guidelines. All 13 target alkaloids displayed excellent linearity within a mass concentration range of 0.2 to 50 ng/mL， with correlation coefficients （*R*^2^） consistently exceeding 0.99. The limits of detection （LODs） and limits of quantification （LOQs）， determined with acceptable accuracy and precision， ranged from 1 µg/kg to 4 µg/kg and 2 µg/kg to 10 µg/kg， respectively. Method accuracy and precision were assessed through recovery tests at three spiking levels， with six replicates at each level. The mean recoveries for all analytes ranged from 83.9% to 119.1%， with associated relative standard deviations （RSDs） all being ≤7.3%， validating the method’s high reliability and repeatability. Systematic evaluation indicated that the matrix effects for the 13 analytes were negligible； hence， the solvent calibration curve was adopted for quantitative analysis. The practical applicability of the validated method was demonstrated through the analysis of 50 batches of commercially available essential oil-based cosmetics. This market survey encompassed 15 products specifically marketed for infants or children and 35 products intended for adult use. As a result， none of the 13 target prohibited alkaloids were detected in any of the tested samples above their respective LOQs. A particularly notable accomplishment of this work is the successful development of a sensitive and reliable quantification strategy for oleandrin， a potent cardiotoxic alkaloid for which standardized detection methods in complex cosmetic matrices such as essential oils have been conspicuously absent. In conclusion， this study successfully establishes a simple， rapid， sensitive， and robust QuEChERS-UPLC-MS/MS method. It is comprehensively validated and clearly well-suited for the routine screening， risk monitoring， and quality control of 13 prohibited alkaloids in a wide array of essential oil-based cosmetics. The method offers reliable technical support for regulatory bodies to enforce safety standards and for manufacturers to ensure the safety and compliance of their products， thereby effectively contributing to the protection of consumer health.

生物碱是广泛存在于自然界植物中的一类含氮有机化合物，具有显著的生物活性^［1］^。医学领域已有多种生物碱因药理作用明确被开发为治疗用药。随着市场对“天然来源”成分的青睐，植物提取物在化妆品中的应用日益广泛，尤其在消费量巨大的精油类产品中，植物活性成分常被用于提升产品功效。然而，植物原料在带来活性成分的同时，也可能引入对人体健康构成威胁的次生代谢产物——生物碱类物质^［2］^。例如，夹竹桃属植物提取物具有抗衰老的功效，但若纯化工艺不当，易引入毒性极强的欧夹竹桃苷。据文献报道，该成分在人体血液中的致死质量浓度约为20 ng/mL^［3］^，其安全风险不容忽视。此外，出于对功效的追求，部分商家甚至违法添加某些禁用生物碱，如具有杀菌除螨作用的西伐丁等甾体类生物碱^［4］^，进一步加剧了化妆品的安全隐患。

为加强监管，国家药品监督管理局在2021年发布的《化妆品禁用原料目录》中已将西伐丁、欧夹竹桃苷等多种生物碱列为禁用成分。然而，目前《化妆品安全技术规范（2015版）》^［5］^及相关增补公告中尚未建立相应的检测方法，导致对该类成分的监管存在技术空白，难以有效保障消费者的用妆安全。因此，研发一种适用于精油类化妆品中多种禁用生物碱的高效、准确检测方法，具有重要的现实意义和紧迫性。

目前，生物碱的检测研究主要集中在食品^［6-8］^、法医毒理^［9-11］^及医药^［12，13］^等领域，在化妆品基质中的分析方法仍较为有限^［2，4，14-16］^，尤其是针对欧夹竹桃苷成分在精油类化妆品中的检测暂未见报道。QuEChERS技术最初由美国农业部Anastassiades教授团队开发^［17］^，旨在实现多种化合物的同步分析与检测。研究发现，该方法在提取极性化合物尤其是碱性化合物方面表现出显著潜力，因而被广泛应用于植物源性样品中农药残留的检测。相较于传统前处理方法，QuEChERS技术具有处理速度快、试剂用量少、回收率高、样品通量大以及成本较低等优势，现已成为样品前处理领域中广泛采用的方法之一。针对前述技术缺口，本研究选取13种潜在风险较高的禁用生物碱（包括西伐丁与欧夹竹桃苷等），将QuEChERS前处理技术与液相色谱-串联质谱分析方法结合，建立了一种适用于复杂精油类化妆品基质的快速、高效检测方法。

## 1 实验部分

### 1.1 主要仪器、材料与试剂

Agilent 1290-6470B 液相色谱-串联质谱系统，配备电喷雾离子源（ESI，美国安捷伦公司）；Heidolph Multi Reax涡旋振荡器（海道尔夫仪器设备（上海）有限公司）；KQ-250台式超声波清洗器（昆山市超声仪器有限公司）；Thermo Fisher LYNX 4000高速落地离心机（美国赛默飞世尔科技公司）。0.22 μm有机尼龙滤膜（上海安谱科学仪器有限公司）。

实验所用化学标准品如下：士的宁（99.9 μg/mL）、番木鳖碱（100.4 μg/mL）、秋水仙碱（99.8 μg/mL）、毛果芸香碱（99.7 μg/mL）、那可丁（100.2 μg/mL）、山梗菜碱（100.0 μg/mL）、阿托品（100.6 μg/mL）、东莨菪碱（100.7 μg/mL）、麻黄碱（100.4 μg/mL）、毒扁豆碱（100.4 μg/mL）、乌头碱（99.8 μg/mL）、西伐丁（99.9 μg/mL）、欧夹竹桃苷（99.5 μg/mL），均购自天津阿尔塔科技有限公司，其中东莨菪碱、山梗菜碱、麻黄碱溶剂为甲醇，其余为乙腈；甲醇、乙腈均为色谱纯，购自霍尼韦尔（中国）有限公司；甲酸为色谱纯，购自德国默克公司；C18粉末购自美国色谱科公司；实验用水为Milli-Q超纯水系统（德国默克公司）制备。

### 1.2 样品前处理

准确称取0.5 g精油类化妆品样品，置于15 mL带盖塑料离心管中，加入5 mL含0.1%甲酸的甲醇提取溶液，涡旋混匀后置于台式超声波清洗器中超声提取5 min。提取完成后，在10 000 r/min的速度下离心5 min，移取1 mL上清液至另一干净离心管中。加入100 mg C18粉末，涡旋混匀以净化提取液，再次以10 000 r/min的速度离心2 min。取上清液经0.22 μm有机尼龙滤膜过滤后，用于后续液相色谱-串联质谱分析。

### 1.3 液相色谱-串联质谱分析

#### 1.3.1 色谱条件

色谱柱：Waters ACQUITY UPLC HSS T3（100 mm×2.1 mm，1.8 μm）。流动相A为乙腈，B为0.1%（体积分数）甲酸水溶液。梯度洗脱程序：0~2 min，5%A~15%A；2~4.5 min，15%A~70%A；4.5~5.5 min，70%A~5%A；5.5~7.0 min，5%A。流速：0.3 mL/min。柱温：30 ℃。进样体积：5 μL。

#### 1.3.2 质谱条件

离子源：电喷雾离子源（ESI^+^模式）；监测方式：多反应监测模式（MRM）；干燥气温度：300 ℃；干燥气流速：9 L/min；雾化气压力：241.32 kPa；鞘气温度：325 ℃；鞘气流速：11 L/min；毛细管电压：4 kV；13种禁用生物碱的监测离子对相关信息见表1。

**表1:** 13种禁用生物碱的保留时间及质谱参数

No.	Compound	Chinese name	*t*_R_/min	Precursor ion （*m*/*z*）	Product ions （*m*/*z*）	Fragmentor/V	Collision energies/eV
1	pilocarpine	毛果芸香碱	2.39	209.3	94.9^*^， 163.5	70	34， 29
2	ephedrine	麻黄碱	2.99	166.1	148.0^*^， 91.1	40	19， 20
3	scopolamine	东莨菪碱	3.56	304.2	138.1^*^， 103.0	110	30， 52
4	eserine	毒扁豆碱	3.72	276.1	162.1^*^， 219.1	60	31， 15
5	brucine	番木鳖碱	3.77	395.1	244.2^*^， 324.3	80	50， 45
6	strychnine	士的宁	3.79	335.2	184.2^*^， 156.2	90	50， 60
7	atropine	阿托品	3.83	290.3	124.1^*^， 93.1	80	35， 42
8	narcotine	那可丁	4.25	414.1	220.0^*^， 353.0	135	20， 24
9	colchicine	秋水仙碱	4.58	400.4	310.1^*^， 358.1	70	35， 32
10	cevadine	西伐丁	4.58	592.4	574.3^*^， 456.2	200	46， 60
11	lobeline	山梗菜碱	4.58	338.3	216.1^*^， 200.1	130	24， 31
12	aconitine	乌头碱	4.70	646.3	586.2^*^， 526.3	140	46， 51
13	oleandrine	欧夹竹桃苷	5.73	577.3	373.3^*^， 112.9	136	14， 30

* Quantitative ion.

### 1.4 标准溶液配制

分别精密量取各生物碱标准品溶液1.0 mL，置于10 mL容量瓶中，用甲醇稀释并定容至刻度，混匀，配制成质量浓度约为10 μg/mL的标准储备液，于4 ℃避光保存，有效期3个月。

临用时，分别精密量取上述各单一标准储备液0.1 mL于同一10 mL容量瓶中，用甲醇稀释并定容，混匀，得到质量浓度均为100 ng/mL的混合标准中间液。

进一步通过精密移取不同体积的混合标准中间液，用甲醇进行逐级稀释，配制成质量浓度范围为0.2~50 ng/mL的系列混合标准工作溶液。所有溶液均需现用现配，待LC-MS/MS分析测定。

## 2 结果与讨论

### 2.1 检测条件的优化

#### 2.1.1 质谱条件的优化

采用质谱仪内置Optimizer软件，对13种生物碱的质谱参数进行系统优化。首先，将质量浓度为100 ng/mL 的混合标准溶液通过两通装置以直接进样方式导入质谱系统。在软件中输入各分析物的精确分子式，设置一级质谱扫描模式为选择离子监测（SIM），设置采集母离子为［M+H］^+^，并自动优化裂解电压（Fragmentor）。在此基础上，进行子离子扫描，通过自动调整碰撞能量（CE），筛选出响应强度最高的两个特征子离子。最后，在MRM模式下，对上述离子对进行碰撞能优化，获得各离子对的最优碰撞能，并获得不同离子对的离子丰度列表。通过以上步骤，获得了13种目标物质的最优定量离子对、定性离子对以及对应的最优碰撞能、裂解电压等质谱参数，具体参数见表1。

#### 2.1.2 色谱条件的优化

为获得最佳分离效果，本实验对比评估了4款不同粒径、长度的色谱柱，包括：Agilent Poroshell 120 C18 （100 mm×3.0 mm，2.7 μm，1号色谱柱）、Phenomenex Kinetex F5（100 mm×3.0 mm，2.6 μm，2号色谱柱）、Agilent RRHD C18 （50 mm×2.1 mm，1.8 μm，3号色谱柱）以及Waters ACQUITY UPLC HSS T3 （100 mm×2.1 mm，1.8 μm，4号色谱柱）。结果表明，1号色谱柱存在峰形拖尾的现象，2号色谱柱的基线波动较为显著，3号色谱柱导致多数目标物保留时间过短、分离度欠佳，而4号色谱柱基线平稳、峰形对称，最为适合，选用为本实验的色谱柱。

在流动相组成方面，本实验分别考察了甲醇、乙腈两种有机相的洗脱效果，结果表明，乙腈作为流动相时，各目标物具有更佳的峰形与更高的质谱响应灵敏度；同时，在水相中添加0.1%（体积分数）甲酸，可有效促进正离子模式下的离子化，增强［M+H］^+^的信号强度。因此，最终确定以0.1%甲酸水溶液和乙腈作为流动相体系，并进一步优化梯度洗脱程序，改善各物质分离效果和质谱响应，优化后的色谱分离结果见图1。

**图1:**
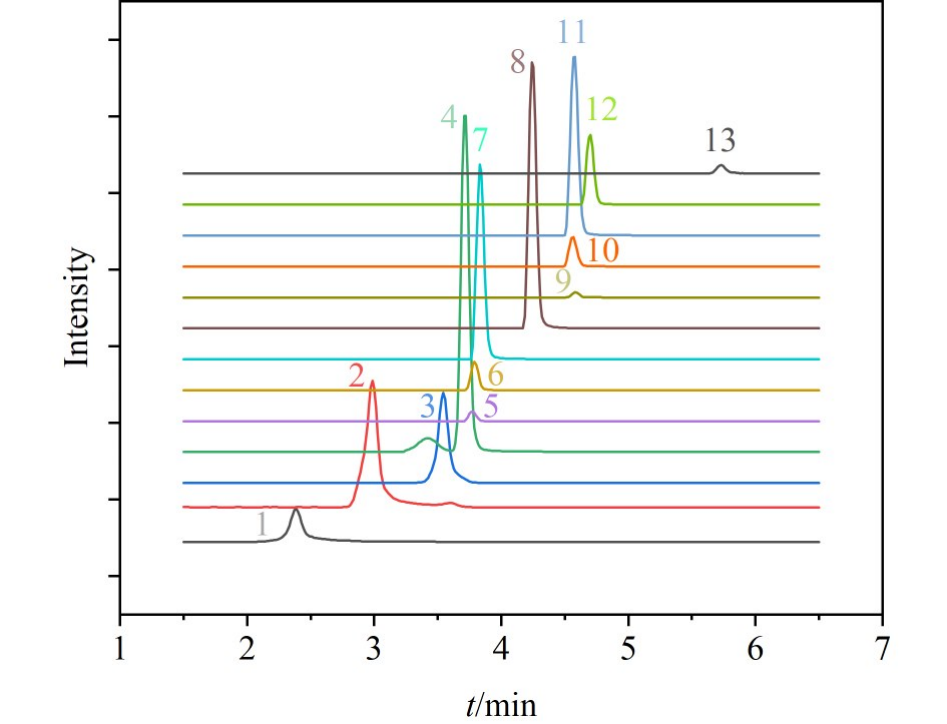
13种禁用生物碱的提取离子流图

### 2.2 前处理实验的优化

#### 2.2.1 提取溶剂的选择

鉴于13种目标生物碱在有机溶剂（如甲醇、乙腈）中具有良好的溶解性，而在水中溶解性欠佳，因此本研究首先采取有机试剂提取方式。通过对比甲醇、乙腈作为溶剂配制的5 ng/mL混合标准溶液的质谱响应，发现甲醇作为溶剂时各目标分析物表现出更好的信噪比（*S/N*）。因此，进一步以加标水平为50 μg/kg的精油类化妆品为对象，系统评价了以不同体积分数（0、25%、50%、75%、100%）甲醇水溶液作为提取溶剂的提取回收率，结果如图2所示。

**图2:**
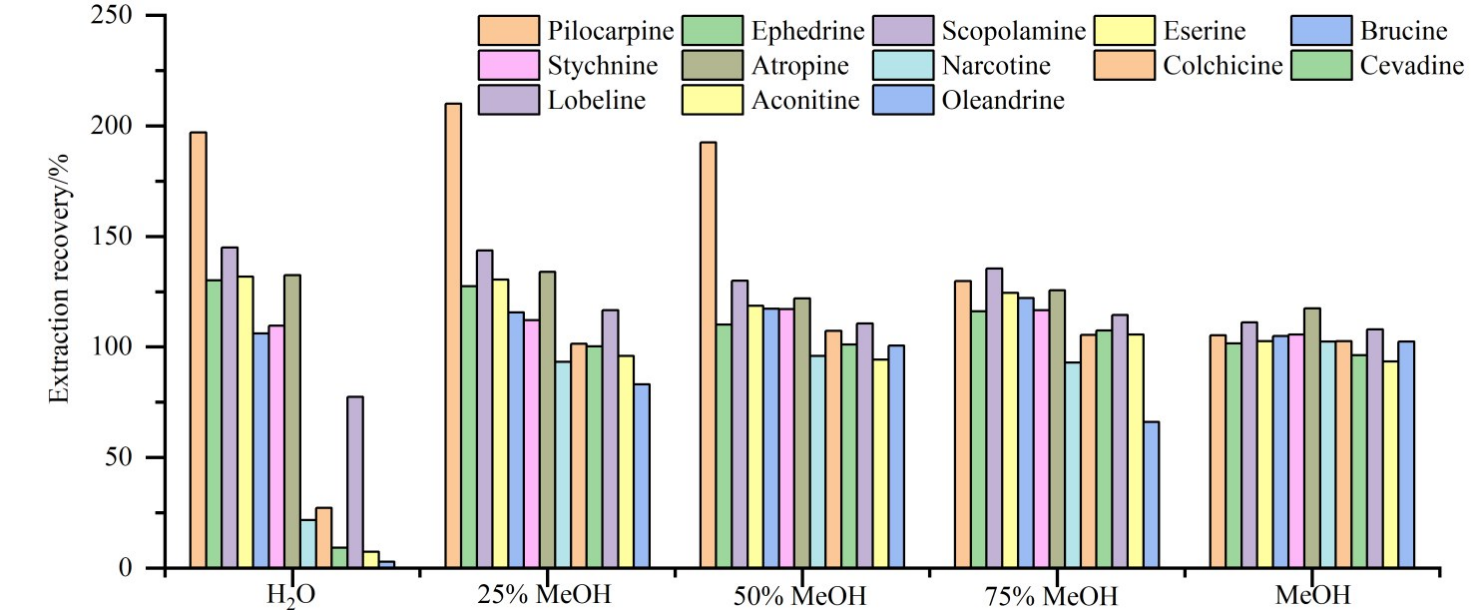
不同体积分数甲醇水溶液对13种禁用生物碱的提取回收率

由图2可见，当提取溶剂中水的比例较高时，毛果芸香碱、麻黄碱等生物碱的提取回收率远远高于100%，这可能由于精油化妆品基质中某些水溶性成分被提取后对目标物产生基质增强效应的影响，而提取溶剂中甲醇含量增加时，各化合物表现出更佳的提取回收率；同时，随着甲醇比例的提升，提取液过有机滤膜时的通滤性显著改善，更有利于后续实验的开展。此外，当使用纯甲醇作为提取溶剂时，离心操作后精油样品位于离心管下层，而上层甲醇提取液澄清，两者分层明显，便于直接取用进行净化，有效简化了前处理流程。因此，最终选择纯甲醇作为提取溶剂。

为进一步提升质谱检测灵敏度，在甲醇提取液中分别添加了0.1%、1.0%和2.0%（体积分数）的甲酸以改善待测液中目标物质的响应。结果发现，过高含量的甲酸会对部分目标物的离子化效率产生抑制效应。综合比较各添加比例下的质谱响应，最终确定添加0.1%甲酸至甲醇提取液中。

#### 2.2.2 净化方式的优化

精油类化妆品是精心调配的工业产品、成分复杂，多含有油脂类物质，易造成色谱柱污染并影响仪器分析性能。为减少基质干扰，本研究对提取液进行进一步的净化处理，以减少油脂类物质的残留。重点比较了QuEChERS方法中常用的几种吸附剂^［17-21］^及液液萃取方式：C18粉末（可以吸附脂类等物质），PSA粉末（能够吸附色素等物质），多壁碳纳米管（MW CNTs），石墨化炭黑粉末（GCB，可以吸附具有平面结构的大极性物质），正己烷液液萃取法。具体操作如下：分别转移1 mL提取液于干净离心管中，加入100 mg上述吸附剂或0.5 mL正己烷，涡旋混合后离心，取上清液进行分析，并比较各目标物的回收率，结果如图3所示。

**图3:**
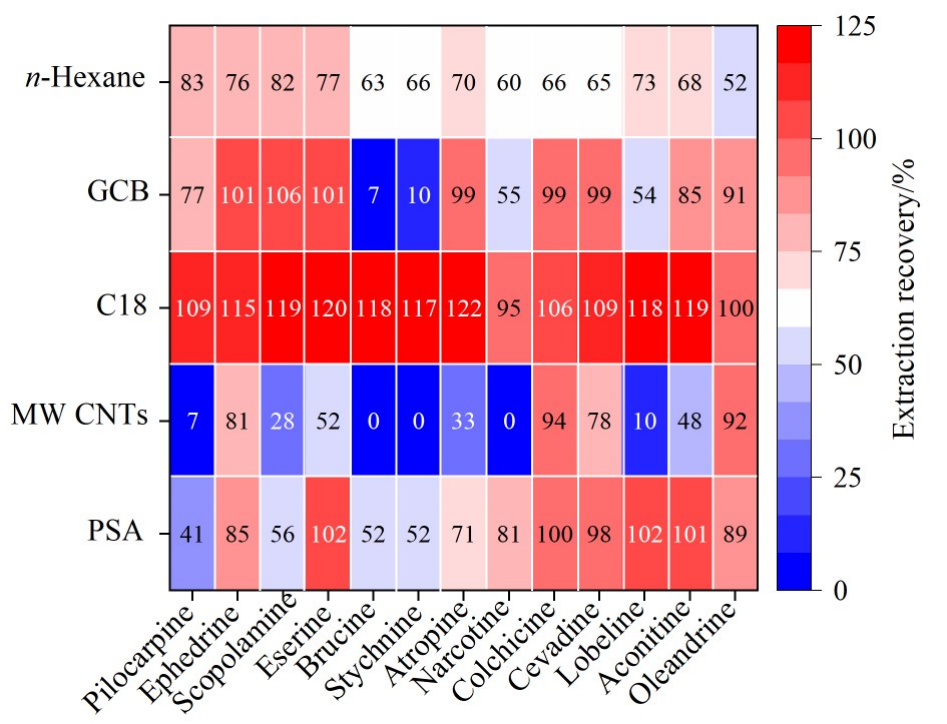
不同净化方式对13种禁用生物碱提取回收率的影响

由图3可知，不同的净化方式对各目标物提取效果影响显著。其中，MW CNTs因与多数目标物分子中的苯环结构存在强烈的*π-π*相互作用，导致目标物被严重吸附，回收率显著降低甚至无法检出。GCB的六元环平面结构使其对那可丁、山梗菜碱、番木鳖碱和士的宁产生强吸附力，尤其是使得后两种生物碱的回收率极低。PSA粉末对部分目标物净化效果良好，但对毛果芸香碱等化合物吸附较强，致其回收率不理想。正己烷萃取净化后各物质回收率表现比较均衡，但普遍偏低，推测正己烷和甲醇存在一定互溶性，导致目标物损失有关。相比之下，使用C18粉末净化时，各目标分析物均能获得较为理想且稳定的回收率。因此，最终选择C18粉末作为QuEChERS净化材料。

### 2.3 基质效应（ME）的评价

由于质谱分析检测时目标物与样品溶液中其他干扰物存在竞争离子源的情形，实验中不能忽视ME的影响。本实验通过对比空白精油基质匹配标准溶液与纯溶剂标准溶液的质谱响应，对13种目标物的基质效应进行了考察。具体操作如下：配制相同质量浓度的精油空白基质标准溶液与溶剂标准溶液，经UPLC-MS/MS分析后，记录各目标物的色谱峰面积，并按式（1）计算ME。

ME=（*A*_0_/*A*_m_-1)×100% （1）

式中，*A*_0_为空白精油基质标准溶液中各物质峰面积，*A*_m_为溶剂标准溶液中各物质峰面积。结果（表2）表明，13种目标物的ME绝对值均在20%以内，表现出较弱的基质效应，实际检测过程中可忽略基质影响。因此采用溶剂标准曲线进行定量分析。

**表2:** 精油基质中13种目标物的线性方程、线性范围、相关系数（*R*^2^）、检出限、定量限及基质效应

Compound	Linear equation	Liner range/（ng/mL）	*R*^2^	LOD/（µg/kg）	LOQ/（µg/kg）	ME/%
Pilocarpine	*y*=3517.49*x*-341.33	0.2-50	0.9969	2	5	6.6
Ephedrine	*y*=13102.25*x*-211.62	0.2-50	0.9995	1	2	-4.3
Scopolamine	*y=*7840.86*x*+613.76	0.2-50	0.9927	1	2	-3.8
Eserine	*y*=20482.57*x*+556.08	0.2-50	0.9982	1	2	-7.6
Brucine	*y*=741.33*x*+24.69	0.2-20	0.9929	2	5	-3.2
Strychnine	*y*=2058.91*x*+85.34	0.2-20	0.9908	2	5	2.2
Atropine	*y*=11534.78*x*+1139.37	0.2-50	0.9926	1	2	1.7
Narcotine	*y*=15954.08*x*+212.84	0.2-50	0.9911	1	2	-1.4
Colchicine	*y*=275.00*x*+5.11	0.2-50	0.9931	2	5	-0.1
Cevadine	*y*=4829.68*x*-23.41	0.2-10	0.9954	2	5	0.9
Lobeline	*y=*10680.48*x*+216.45	0.2-50	0.9964	1	2	-3.2
Aconitine	*y*=5146.88*x*+2.62	0.2-20	0.9973	2	5	-8.9
Oleandrine	*y*=66.59*x*+0.51	0.2-20	0.9981	4	10	2.6

*y*： peak area； *x*： mass concentration， ng/mL.

### 2.4 方法学考察

#### 2.4.1 线性关系及定量限

在1.3节条件下，对1.4节配制的系列混合标准工作溶液进行测定。以各目标物的质量浓度、峰面积分别为横、纵坐标绘制标准曲线。结果如表2所示，13种生物碱在考察范围内均呈现良好的线性关系。

在空白精油基质中添加13种禁用生物碱混合标准溶液并逐级稀释，以定量离子的*S/N*不低于3为判定标准，确定各物质的检出限（LOD），并以定量离子的*S/N*不低于10为判定标准，确定各物质的定量限（LOQ），结果见表2。

#### 2.4.2 加标回收试验

为评估方法的准确度与精密度，在空白精油基质中分别按照1倍定量限（1 LOQ）、2倍定量限（2 LOQ）和10倍定量限（10 LOQ）进行3个水平的加标回收试验，每个水平平行制备 6 份样品，结果（见表3）表明，精油中13种目标物的平均回收率范围为83.9%~119.1%，RSD≤7.3%，表明本方法具有良好的准确度和重复性，能够满足精油样品中目标物定量分析的要求。

**表3:** 13种禁用生物碱在3个水平下的加标回收率和RSD（*n*=6）

Compound	1 LOQ		2 LOQ		10 LOQ	
	Recovery/%	RSD/%	Recovery/%	RSD/%	Recovery/%	RSD/%
Pilocarpine	87.4	6.2	88.8	2.7	108.7	5.5
Ephedrine	85.8	5.4	102.5	3.6	116.0	0.2
Scopolamine	83.9	7.3	106.5	3.3	99.3	2.2
Eserine	84.5	5.9	105.7	4.4	98.4	1.1
Brucine	87.5	5.5	97.5	4.3	117.7	4.4
Strychnine	92.5	5.2	119.1	4.5	117.0	6.1
Atropine	90.3	6.4	101.9	4.8	96.5	3.2
Narcotine	92.2	3.9	90.7	3.9	99.6	5.8
Colchicine	89.2	6.7	104.7	5.0	106.4	4.2
Cevadine	94.0	6.9	99.5	5.7	109.5	4.2
Lobeline	93.1	6.3	103.6	2.6	98.9	6.9
Aconitine	101.4	4.8	88.6	7.1	119.0	3.4
Oleandrine	93.0	3.3	101.4	1.5	103.5	2.2

### 2.5 实际样品的检测

采用本方法对50批市场购买的精油类化妆品进行检测，其中15批为宣称婴幼儿使用或儿童产品，35批为成人用精油产品。结果显示，所有样品中均未检出本方法所涵盖的13种禁用生物碱。

为验证方法对实际阳性样品的检测能力，随机选取1批宣称婴幼儿使用甘油，加入欧夹竹桃苷标准溶液后模拟阳性样品进行检测，测得回收率为99.6%，进一步证实了本方法在实际应用中的可靠性。其典型MRM色谱图见图4。

**图4:**
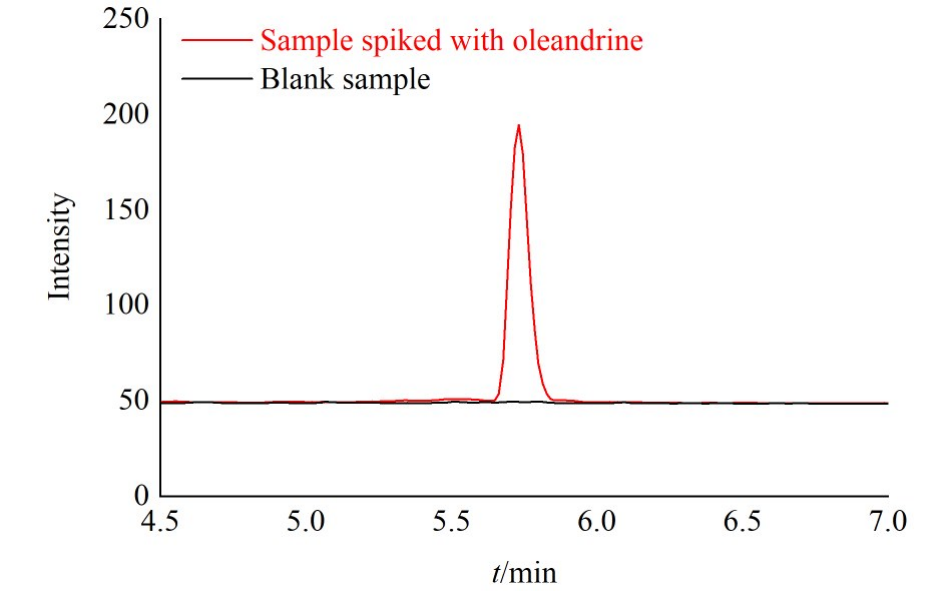
欧夹竹桃苷阴性空白样品及模拟阳性样品的MRM色谱图

## 3 结论

本研究成功建立了一种基于QuEChERS前处理技术与液相色谱-串联质谱联用的分析方法，可同时测定精油类化妆品中13种禁用生物碱。该方法操作简便，净化效果好，分析周期短，重复性好，能够实现对13种目标物的同步定性和定量分析，满足批量样品的快速检测需求。经验证，方法在线性范围、灵敏度、准确度与精密度方面均表现良好，且基质效应不显著，可采用溶剂标准曲线进行准确定量。

特别地，本方法实现了对精油类化妆品中欧夹竹桃苷的快速、灵敏定量，该检测方法的建立为该领域检测技术的发展提供了新的思路。所建立的方法可为相关监管工作和企业质控提供可靠的技术支撑，从而助力化妆品安全风险的早期识别与有效防控。
